# Estimation of basic reproduction number (R_0_) of African swine fever (ASF) in mid-size commercial pig farms in Vietnam

**DOI:** 10.3389/fvets.2022.918438

**Published:** 2022-09-29

**Authors:** Nguyen Tuan Anh Mai, Thi Bich Ngoc Trinh, Van Tam Nguyen, Thi Ngoc Ha Lai, Nam Phuong Le, Thi Thu Huyen Nguyen, Thi Lan Nguyen, Aruna Ambagala, Duc Luc Do, Van Phan Le

**Affiliations:** ^1^College of Veterinary Medicine, Vietnam National University of Agriculture, Hanoi, Vietnam; ^2^Animal Science and Veterinary Medicine Faculty, Bac Giang Agriculture and Forestry University, Bac Giang, Vietnam; ^3^National Centre for Foreign Animal Disease, Canadian Food Inspection Agency, Winnipeg, MB, Canada; ^4^College of Animal Sciences, Vietnam National University of Agriculture, Hanoi, Vietnam

**Keywords:** African swine fever, African swine fever virus, basic reproduction number, epidemiology, African swine fever (ASF) decision making

## Abstract

African swine fever (ASF) is a devastating disease affecting the global swine industry. Recently, it has spread to many countries in Africa, Europe, Asia, and the Caribbean, leaving severe damage to local, regional, national, and global economies. Due to its highly complex molecular characteristics and pathogenesis, the development of a successful vaccine has been an unmet challenge. Therefore, ASF control relies solely on biosecurity, rapid detection, and elimination. Epidemiological information obtained from natural ASF outbreaks is critical for designing and implementing ASF control measures. Basic reproduction number (R_0_), an epidemiological metric used to describe the contagiousness or transmissibility of infectious agents, is an important epidemiological tool. In this study, we have calculated R_0_ for the in-farm spread of ASF among fattening pigs and sows in two midsize commercial pig farms, HY1 and HY2, that practice the spot removal approach in controlling ASF outbreaks in Vietnam. The R_0_ values for the sows and fattening pigs were 1.78 (1.35–2.35) and 4.76 (4.18–5.38) for HY1 and 1.55 (1.08–2.18) and 3.8 (3.33–4.28) for HY2. This is the first study to evaluate the transmission potential of ASF in midsize commercial pig farms in Vietnam. Based on the R_0_ values, we predict that the spot removal approach could be used to successfully control ASF outbreaks in midsize commercial sow barns but not in fattening pens.

## Introduction

African swine fever (ASF) is one of the most dangerous infectious diseases of swine and causes nearly 100% mortality in infected animals. It was first reported in Kenya in 1921 and recognized as an endemic disease in Sub-Saharan Africa (1). In 2007, Georgia reported its first outbreak, followed by epidemics in Russian Federation, Caucasus, Belarus, and Ukraine (2). On 1 August 2018, ASF was confirmed in a pig farm in Shenbei district of Shenyang, Liaoning province, China. The outbreak killed 47 out of 383 pigs in the farm. Later, it was confirmed that the ASF virus (ASFV) responsible for the outbreak belonged to p72 genotype II, closely related to the virus circulating in Europe and the Russian Federation (3). Subsequently, ~165 ASF outbreaks were reported in 32 provinces in China that killed over one million pigs (http://www.fao.org/ag/againfo/programmes/en/empres/ASF/situation_update.html). In early February 2019, the first outbreak of ASF in Vietnam was reported in Hung Yen province (4). Then, it quickly spread to the rest of the country and affected all 63 provinces. Over six million pigs were killed in the process to stop the disease and control the situation (FAO and Ministry of Agriculture and Rural Development, Vietnam). ASF is now considered endemic in many countries in Southeast Asia including Vietnam. There have been many attempts to develop an effective vaccine for ASF with limited success (5, 6). At present, the only viable strategy for ASF eradication is by stamping it out.

The majority of commercial pig farms in Vietnam are midsize farms that house several hundreds to thousands of pigs. They are operated independently by farmers or under contracts with private companies. At the beginning of the ASF outbreak in Vietnam, rapid detection and complete depopulation of commercial pig farms were employed. This approach, together with the high mortality associated with ASF, led to rapid depletion of the national swine population in Vietnam and severe economic burden on pig farmers. Therefore, the Department of Animal Health in Vietnam allowed practicing spot elimination, which is rapid detection and removal of only ASFV-infected animals (also called “pulling the tooth”). The success of this method depends on many factors including contagiousness of the ASF virus responsible, veterinary infrastructure, sound and readily accessible veterinary diagnostics, strong biosecurity practices, and epidemiological situation of the disease in the affected region.

Basic reproduction number (R_0_), the number of secondary cases generated from a single infected individual in a susceptible population, is a critical epidemiological tool (7–10). In addition, basic reproduction number (R_0_) represents the total counting of the number of generated secondary cases for the entire period of the infection of the initial case. It provides information required to understand outbreak dynamics and the scale speed of disease spread. It is useful for evaluating potential disease control strategies (11). R_0_ is not a biological constant for a given pathogen, and it is affected by a number of geographical and epidemiological factors such as types of pig (domestic vs. wild boars), farm type (backyard vs. commercial), size, biosecurity, and sanitary levels of the affected farms (12–15). The aim of this study was to provide an estimated R_0_ value calculated based on the information obtained from two midsize commercial pig farms in Vietnam that conduct spot elimination.

## Methods

### Farm design and capacity

For this study, two commercial farrow-to-finish pig farms (HY1 and HY2) located in two different districts in Hung Yen province, Vietnam were selected immediately after ASF outbreaks were confirmed in the two farms. The two farms belonged to two different private companies. The farms recorded and reported the daily status of herds since the R_0_ values of commercial farms are limited in terms of epidemiology. Therefore, we chose the two farms to obtain more information on disease progression inside restricted facilities. Both farms use the close-system model and are designed according to the standard commercial swine barn layout in which sows are housed individually in single stalls and fattening pigs in groups of 30-40 per pen. Both farms are equipped with automatic cooling systems, and the sows and fattening pigs are housed 50-100 m apart from each other. The ages of the fattening pigs ranged from 10 to 22 weeks. The capacity and the total number of pigs in each farm are shown in Supplementary Table S1. Strict biosecurity measures, daily cleaning, and rapid disposal of sick/dead animals followed by thorough disinfection are practiced in both farms (Supplementary Table S2). In addition, both farms use commercial grade rations from different suppliers and practice no swill feeding; the workers are assigned to each individual barn, and no visitors are allowed in the farms.

### Data source

The ASF outbreak in each farm was confirmed by real-time PCR (VDx^®^ ASFV qPCR; Median Diagnostics Inc., Seoul, Korea) as described previously (16) using whole blood samples collected from pigs showing fever (rectal temperatures above 40°C for more than 2 days), loss of appetite, and/or cutaneous hemorrhages. Data related to each farm and the ASF outbreak were collected from the respective farm owners. Since determining the exact initial day of the ASF infection was not possible, the first day each farmer noticed the above clinical signs was considered the initial day of infection, and the day the whole herd was culled was the end of infection (15). During the study, the pigs in both affected farms were monitored daily for clinical signs. Whole blood was collected from any animal showing ASF-like clinical signs and tested for ASF by real-time PCR.

### Definitions

A confirmed case of ASF was defined as pigs showing high fever, anorexia, lethargy, cutaneous hemorrhages, or death followed by a positive ASFV real-time PCR result. The serial interval was defined as the time gap between the onset of the primary and secondary cases in the chain of transmission.

### Statistical analysis

In this study, we used the R programming language (version 4.0.5, https://www.r-project.org/about.html) to perform statistical analysis. We assumed that any pig showing ASF-like symptoms for the first time and was later confirmed by real-time PCR as an infected case. For each farm, the basic reproduction number (R_0_/R naught) was calculated for sows and fattening pigs separately using the maximum likelihood method in “earlyR”. The package “projections” was used to produce a plausible trajectory prediction of newly infected cases and cumulated cases of each outbreak in the next 14 days (17–20). The mean and standard deviation of the deaths were used to estimate R_0_ and fitted in a gamma distribution. The maximum likelihood method and “get_R” function were used to calculate R_0_ distribution. The likely values of R_0_ were generated using the bootstrap method with 1,000 replicates and presented in a histogram format. The prediction and simulation require the existing daily incidence, a serial interval distribution, and the estimated R_0_ values under the assumption that they are being fitted into the Poisson distribution and based on the daily record of infected cases.

### Early R mathematical model


$$R_{0}=\sum_{\text{s}=1}^{\text{t}}\text{I}\left(\text{t}-\text{s}\right)\text{ws}$$

The ratio of the number of newly infected cases created at time step t, It, to the total infectiousness of infected cases at time t, provided by the sum of infection incidence up to time step t-1, weighted by the infectivity function ws, is used to calculate estimated R_0_. If the circumstances stayed the same at time t, each sick individual would infect an average of R_0_ secondary cases (18).

### Projections mathematical model

We fit the data of estimated R_0_, daily incidence, and a serial interval into the model, which is denoted as:


$$\begin{array}{l} \lambda t=\;\overset{t-1}{\underset{s=1}{\sum }}ysw\;\left(t-s\right) \end{array}$$

where *ys* is the incidence in the real-time event at time *S* and *w (t–s)* is the probability mass function vector of serial interval distribution. The model is based on the assumption that daily incidence carries out by approximately Poisson distribution when daily infectiousness can be determined (20).

## Results

**Figure 1:**
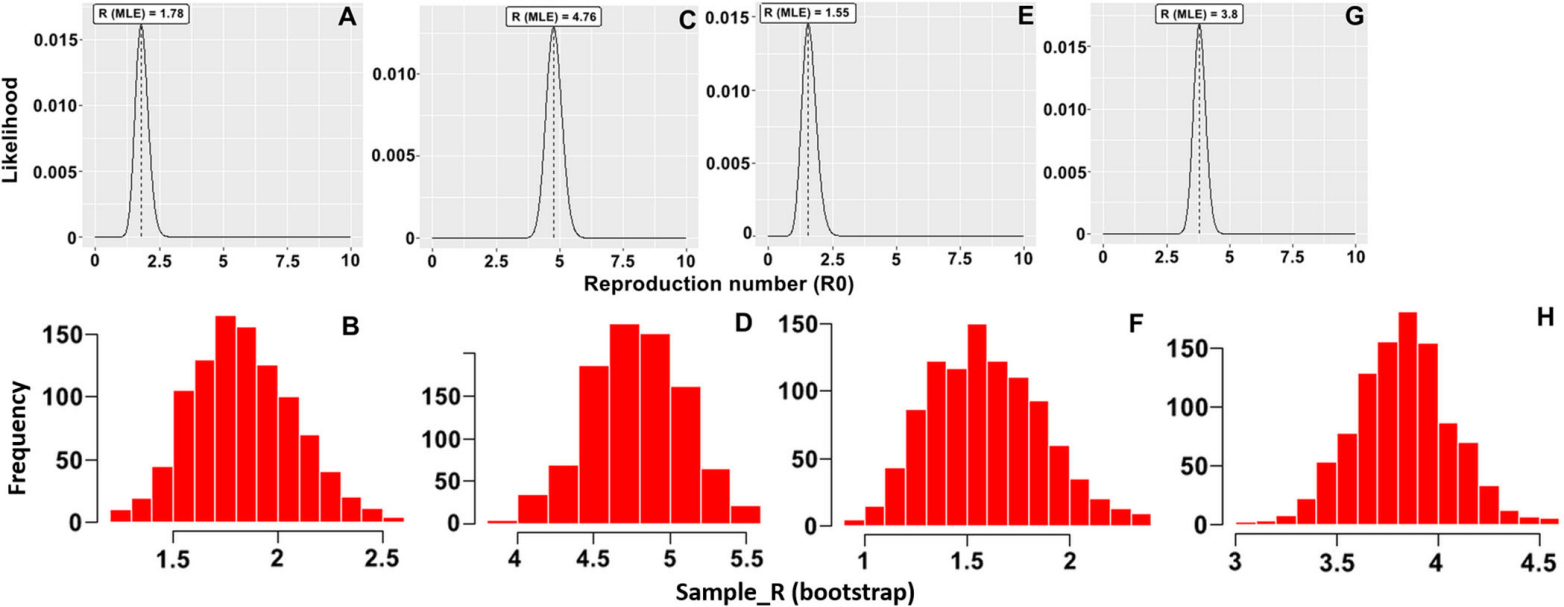
Distribution of likely R_0_ value with the maximum likelihood (ML) method and histogram of 1,000 likely R_0_ values using the bootstrap method for **(A,B)** sows and **(C,D)** fattening pigs in the HY1 farm and **(E,F)** sows and **(G,H)** fattening pigs in the HY2 farm.

**Figure 2:**
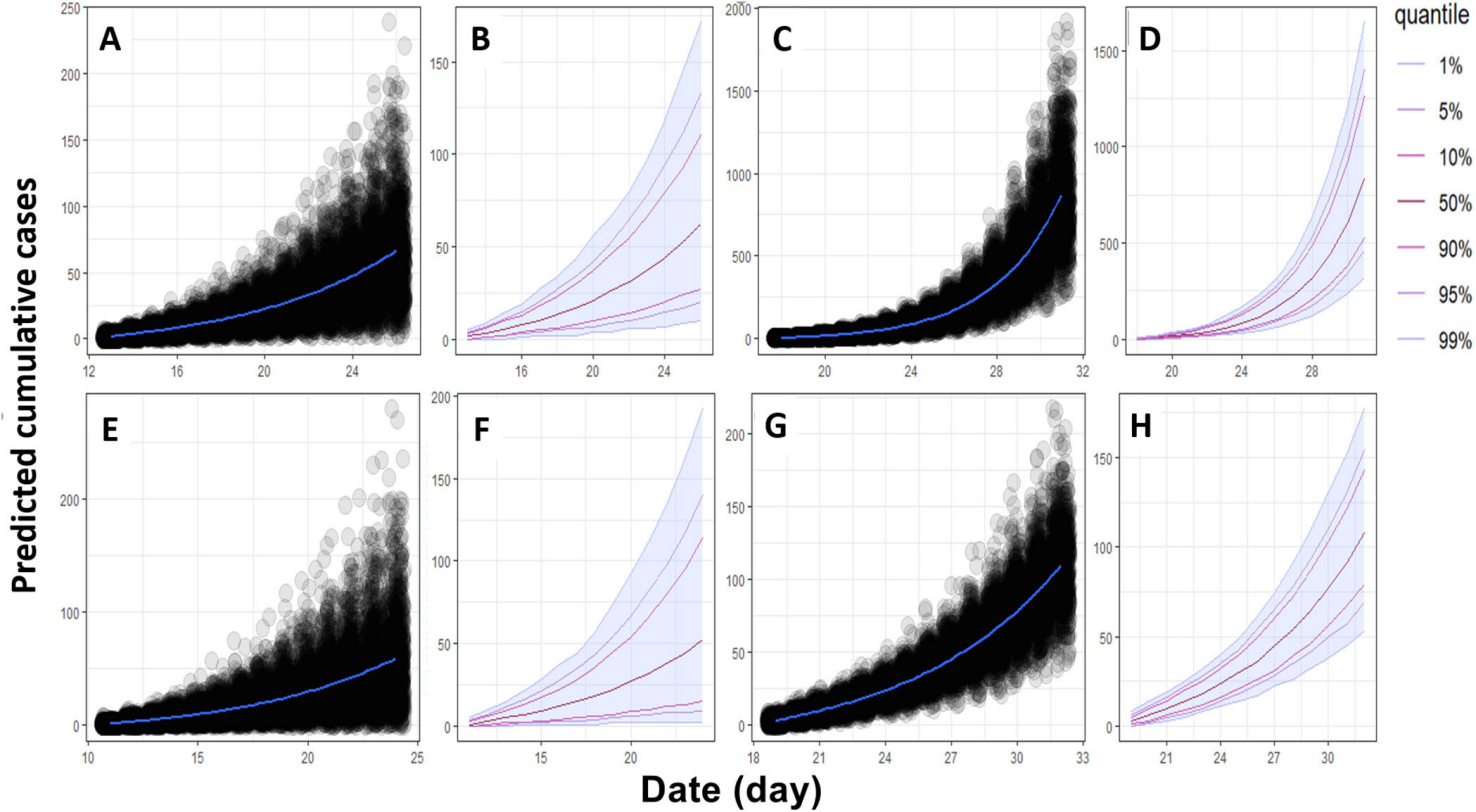
Epidemiological trajectories of expected new cumulative cases of ASF in both farms in the next 14 days. **(A,B)** new cumulative cases in sows and **(C,D)** new cumulative cases in fattening pigs in the HY1 farm. **(E,F)** new cumulative cases in sows and **(G,H)** new cumulative cases in fattening pigs in the HY2 farm.

The basic reproduction number (R_0_) of an infectious pathogen is the average number of infected cases directly generated by one case in a population. Previous studies have shown that the R_0_ values for ASFV generated from domestic pigs and wild boars in the field were different from those measured under experimental conditions (13–15, 21, 22). In this study, we report the in-farm R_0_ for sows and fattening pigs in two midsize commercial farms in Vietnam. The maximum likelihood method (Figures 1A,C,E,G) was used to produce R_0_ estimates for HY1 sows (1.78) and fattening pigs (4.76) and HY2 sows (1.55) and fattening pigs (3.8). The bootstrap method (Figures 1B,D,F,H) was used to estimate R_0_ values after fitting the collected data to the Poisson distribution. During each outbreak in the study, the mean infected cases of sows and fattening pigs per day were 4.5 and 13.94 for HY1 and 3.3 and 14.28 for HY2 (Table 1). The in-farm R_0_ estimated with a 95% confident interval (C.I) for the sows and fattening pigs was 1.78 (1.35–2.35) and 4.76 (4.18–5.38) the HY1 and 1.55 (1.08–2.18) and 3.8 (3.33–4.28) for HY2 (Table 1 and Figures 1A,C,E,G). Using the R_0_ values, the probable and plausible number of new cases in each farm for the subsequent 14 days was calculated using the “projections” package (Table 2 and Figure 2). Based on the calculation, the cumulative cases for HY1 for the subsequent 14 days were 17.45 and 51.55% for the sows and fattening pigs, respectively. For HY2, the predicted cumulative cases were 30.73% for the sows and 11.21% for the fattening pigs.

**Table 1:** Mean, the standard deviation of infected cases per day, and R_0_ values.

**Farm**	**Type of pig**	**Actual pig population**	**Mean**	**Standard deviation**	**Basic reproduction number (R_0_) (95% C.I)**
HY1	Sow	384	4.5	2.78	1.78 (1.35–2.35)
	Fattening	1682	13.94	15.98	4.76 (4.18–5.38)
HY2	Sow	192	3.3	2.54	1.55 (1.08–2.18)
	Fattening	981	14.28	10.25	3.80 (3.33–4.28)

CI, Confident interval.

**Table 2:** Prediction of daily and cumulative cases for the next 14 days based on the obtained R_0_.

**Day**	**HY1**				**HY2**			
	**Cases per day**		**Cumulated cases**		**Cases per day**		**Cumulated cases**	
	**Sow**	**Fattening**	**Sow**	**Fattening**	**Sow**	**Fattening**	**Sow**	**Fattening**
1	2 (0–4)	4 (1–8)	2 (0–5)	4 (1–8)	1 (0–4)	3 (0–7)	1 (0–4)	3 (0–7)
2	2 (0–5)	5 (1–11)	4 (0–8)	9 (3–18)	2 (0–5)	3 (0–7)	3 (0–8)	6 (2–12)
3	2 (0–6)	7 (2–14)	6 (1–12)	17 (6–31)	2 (0–6)	4 (1–8)	5 (0–12)	10 (4–18)
4	2 (0–7)	10 (4–19)	8 (2–17)	27 (11–49)	2 (0–7)	4 (0–9)	7 (1–18)	14 (6–24)
5	3 (0–7)	14 (5–26)	11 (3–22)	42 (18–72)	2 (0–8)	4 (1–9)	10 (1–23)	19 (10–30)
6	3 (0–8)	19 (7–36)	14 (3–29)	61 (28–106)	3 (0–9)	5 (1–10)	13 (2–31)	24 (13–37)
7	4 (0–10)	26 (10–46)	18 (5–38)	88 (40–151)	3 (0–11)	6 (1–12)	16 (2–40)	30 (16–47)
8	4 (0–11)	36 (14–64)	22 (6–47)	124 (57–214)	4 (0–13)	7 (2–14)	20 (3–52)	37 (21–58)
9	5 (0–13)	48 (20–84)	28 (7–59)	173 (78–300)	5 (0–14)	8 (3–15)	24 (3–65)	45 (26–69)
10	5 (0–14)	65 (27–114)	33 (8–72)	241 (110–421)	5 (0–17)	9 (3–17)	30 (4–79)	55 (31–83)
11	6 (1–17)	89 (38–160)	40 (9–88)	334 (149–571)	6 (0–19)	11 (4–20)	36 (4–95)	66 (37–99)
12	7 (0–18)	123 (53–217)	47 (10–106)	461 (212–787)	7 (0–22)	13 (5–22)	42 (4–113)	78 (44–118)
13	8 (1–20)	167 (72–295)	56 (12–128)	633 (290–1073)	8 (0–26)	14 (6–25)	50 (5–140)	93 (52–140)
14	10 (1–24)	227 (99–401)	67 (14–156)	867 (398–1476)	9 (0–29)	17 (7–29)	59 (5–166)	110 (62–165)

## Discussion

The two farms enrolled in this study ended up eliminating their entire herd within 18 days since the first detected case. By the time of stamping out, ASF had claimed the lives of 14.06% (54/384) of the sows and 14.09% (237/1682) of the fattening pigs in HY1, and 17.19% (33/192) of the sows and 26.2% (257/981) of the fattening pigs in HY2 (Supplementary Table S1). R0 value is not a biological constant for a specific pathogen, and it is affected by many factors such as infectiousness of the ASFV strain, duration of infectivity of affected pigs, number of susceptible pigs in the farm, types of pig (domestic vs. wild boars), type of farm (backyard vs. commercial), biosecurity, and sanitary levels of the affected farm (12–15). The infection status of ASF-infected pigs is generally unknown in the field, and R_0_ values are calculated based on the specific group of pigs under consideration. Therefore, R_0_ estimates are also dependent on how the population at risk is defined and how large it is. Therefore, a comparison of R_0_ estimated from different studies is challenging. Within farm R_0_, the values calculated in our study for fattening pigs (4.76 for HY1 and 3.8 for the HY2) were lower than what was reported for fattening pigs under experimental conditions and in some natural outbreaks. Within farm R_0_, the values calculated for a historic outbreak of ASF genotype I in Ukraine in 1977 ranged from 5.68 to 9.21 (21). The R_0_ values calculated under experimental conditions and using a moderately virulent strain ASFV Malta 78 ranged from 6.9 to 46.9 (22). The lower R_0_ values observed in our study for fattening pigs could be due to many factors including the strict biosecurity measures when the first case was reported, daily cleaning, and spot removal followed by thorough disinfection deployed in both ASF-infected farms.

The calculated R_0_ values of the sows in both farms in our study (1.78 for HY1 and 1.55 for HY2) were significantly lower. This could be due to many factors including better management, sanitation, biosecurity conditions in the midsize farms, and the spot removal approach that quickly removed infected animals from the farms. The closest match for our R_0_ values for the sows is the R_0_ values calculated for wild boars in the Czech Republic (R_0_ = 1.95) and Belgium (R_0_ = 1.65). In both of these studies, R_0_ was calculated based on the identification of fresh carcasses of dead wild boars recovered in infected zones (15). Moreover, the accuracy of the R_0_ value relies mostly on whether all infected cases have been identified. All the pigs showing symptoms underwent a real-time PCR test. Therefore, the percentage of unidentified cases is considered low. We suspect that the low R_0_ values in our study may be highly affected by the improved biosecurity of the farms and the fact that all the herds were culled, which may not represent the true progression of an outbreak.

ASF has been endemic in the domestic pig population of Vietnam, and under the current ASF situation and its control strategy in Vietnam, almost all commercial pig farms apply higher biosecurity levels as described in Supplementary Table S2. Once ASF is confirmed in a sow farm, farmers quickly apply the spot removal strategy by removing sick/dead sows and two adjacent sows, followed by thorough cleaning and disinfection of the farm, equipment, etc. (Supplementary Table S2). At the same time, the farmer will also reduce the density of the sows on the farm by the removal of the weak, old, and reproductive impairment/failure sows. In the case of the fattening pig farms, when the first case of ASF was confirmed in the pen, the farmers quickly remove all the pigs in the infected and two adjacent pens, followed by thorough cleaning and disinfection of the farm, equipment, etc.

Despite these efforts, as to what were seen in the HY1 and HY2 farms, ASF continued to spread in some commercial farms in Vietnam. The success of spot removal depends on many factors, including the time taken to detect ASF in a given farm, the biosecurity level of the farm, the experience of the farm crew in handling infectious diseases in pigs, etc. For the fattening pig farms in Vietnam, the spot removal approach appears to only reduce the speed of transmission in the farms, but ultimately most of the farms stamped out the whole herd. This is supported by the calculated R_0_ values in this study. It is accepted that an infectious disease outbreak ends if the R_0_ value is < 1, and it continues if R_0_ has a value > 1 (23). Therefore, for fattening pigs, with R_0_ values ranging from 3.33 to 5.38, we suggest that spot removal is highly unlikely to work; therefore, culling the entire herd is the best option. In contrast, in sow farms in which pigs are individually housed and high biosecurity and management practices are implemented, R_0_ can be brought under 1. In the HY1 and HY2 farms used in our study, the R_0_ values calculated ranged from 1.08 to 2.35. Therefore, with further improvements in the detection and removal of sick/infected animals and additional biosecurity measures, spot removal could be performed to control ASF infections in breeding farms. This will avoid the total depopulation of highly valuable sow farms and, in turn, shortage of piglets. In line with this, observations from field veterinarians show that most midsize sow farms in Vietnam that enforce high biosecurity measures and spot removal are able to quickly and successfully eradicate ASF outbreaks (personal communication with swine veterinarians in Vietnam).

For further prediction of new cumulative cases in the next 14 days, the results were 17.45 and 51.55% for the sows and fattening pigs in the HY1 farm, respectively. For HY2, the predicted cumulative cases were 30.73% for the sows and 11.21% for the fattening pigs. The prediction of new cumulative cases of the fattening pigs in the HY2 farm was the lowest (11.21%) despite the high R0 value (R_0_ = 3.8). The prediction model highly depends on the daily cumulative cases following an exponential trend, which is directly affected by the improved biosecurity measures implemented by the farms. The model performs best when the near-future patterns of incidence follow an exponential trend. However, cumulative cases of the HY2 fattening pigs partially followed an exponential pattern because the reported cases did not represent an entire transmission process but only its early stage. The differences between the predicted and field data were a drawback of this model, as discussed in a previous study (19).

In conclusion, in this study, we calculated within farm R_0_ values for two ASF-affected midsize commercial farms in Vietnam that practiced spot removal to control the spread of the outbreak. Both farms failed to completely stop the spread of ASF and ultimately were depopulated. Based on the R_0_ values calculated in this study, it was evident that spot removal of fattening pigs is highly unlikely to be successful. However, with additional improvements in the area of veterinary oversight on identification, laboratory confirmation, rapid removal, disposal of infected animals, and additional biosecurity measures, spot removal may be a practical approach for sow farmers to successfully control ASF outbreaks. The R_0_ estimations calculated in this study can also be used for other ASF-related epidemiological studies on midsize commercial pig farms in Vietnam and other countries.

## Data availability statement

The original contributions presented in the study are included in the article/Supplementary material, further inquiries can be directed to the corresponding authors.

## Author contributions

AA, DD, VL, and TLN supervised and suggested experiment ideas. NL, TTHN, TL, and VN collected the field samples and daily farm data. NM and TT analyzed the data and prepared the manuscript. The final manuscript was approved by all authors.

## Funding

This study was funded by the Ministry of Agriculture and Rural Development under the project entitled Study on natural resistance to African swine fever of surviving pigs in outbreak areas in Vietnam.

## Conflict of interest

The authors declare that the research was conducted in the absence of any commercial or financial relationships that could be construed as a potential conflict of interest.

## Publisher's note

All claims expressed in this article are solely those of the authors and do not necessarily represent those of their affiliated organizations, or those of the publisher, the editors and the reviewers. Any product that may be evaluated in this article, or claim that may be made by its manufacturer, is not guaranteed or endorsed by the publisher.
