# Supplementary material for: Estimation of basic reproduction number (R0) of African swine fever (ASF) in mid-size commercial pig farms in Vietnam
Source: Front Vet Sci. 2022 Sep 29;9:918438. doi: 10.3389/fvets.2022.918438 (PMC9556723; doi:10.3389/fvets.2022.918438)
Supplement: Supplementary file 2 [file Table_2.DOCX]

| **Supplement Table 2**. Biosecurity measures applied on the two commercial farms |
| --- |

1. Sows and fattening pigs are housed in separate buildings
2. Pigs are kept in a closed system (the air is filtered)
3. A Biogas system is used to dispose of animal waste
4. Water tanks are treated with Chlorine for microbe management
5. Vehicles must pass through security gates
6. Before entering or leaving the farm, vehicles must be sterilized at the stop station.
7. Powdered lime is sprayed on the floor of every gate before and after the vehicles go through.
8. Workers must follow sterilization protocol before entering or leaving the farm.
9. Daily cleaning and disinfection of the farm using disinfectants
10. Every personal equipment must be sterilized under UV light for 30 minutes daily
11. Sterile needles and syringes are used throughout the farm
12. No pig meat or pork products are allowed on the farm
13. Swill feeding is prohibited
14. Rat, fly, and mosquito controls are in place
15. Raising other animals (dogs, cats, etc.) is forbidden
16. Disinfectant Virkon S is used at 1/500 concentration
